# A Narrative Review of the Dorsal Root Ganglia and Spinal Cord Mechanisms of Action of Neuromodulation Therapies in Neuropathic Pain

**DOI:** 10.3390/brainsci14060589

**Published:** 2024-06-09

**Authors:** Matheus Deroco Veloso da Silva, Geovana Martelossi-Cebinelli, Kelly Megumi Yaekashi, Thacyana T. Carvalho, Sergio M. Borghi, Rubia Casagrande, Waldiceu A. Verri

**Affiliations:** 1 Laboratory of Pain, Inflammation, Neuropathy and Cancer, Department of Immunology, Parasitology and General Pathology, Londrina State University, Londrina 86057-970, PR, Brazil; matheus.deroco@gmail.com (M.D.V.d.S.); geovana.martelossi@uel.br (G.M.-C.); kelly.megumi.yaekashi@uel.br (K.M.Y.); sergio.borghi@cogna.com.br (S.M.B.); 2 Department of Pediatrics, Division of Infectious Diseases and Immunology, Guerin Children’s at Cedars-Sinai Medical Center, Los Angeles, CA 90048, USA; thacyana.teixeiradecarvalho@cshs.org; 3 Center for Research in Health Sciences, University of Northern Paraná, Londrina 86041-140, PR, Brazil; 4 Department of Pharmaceutical Sciences, Center of Health Science, Londrina State University, Londrina 86038-440, PR, Brazil; rubiacasa@uel.br; 5 Biological Sciences Center, State University of Londrina, Rod. Celso Garcia Cid Pr 445, KM 380, P.O. Box 10.011, Londrina 86057-970, PR, Brazil

**Keywords:** neuropathic pain, neuromodulation, central sensitization, glial cells, current treatment

## Abstract

Neuropathic pain arises from injuries to the nervous system in diseases such as diabetes, infections, toxicity, and traumas. The underlying mechanism of neuropathic pain involves peripheral and central pathological modifications. Peripheral mechanisms entail nerve damage, leading to neuronal hypersensitivity and ectopic action potentials. Central sensitization involves a neuropathological process with increased responsiveness of the nociceptive neurons in the central nervous system (CNS) to their normal or subthreshold input due to persistent stimuli, leading to sustained electrical discharge, synaptic plasticity, and aberrant processing in the CNS. Current treatments, both pharmacological and non-pharmacological, aim to alleviate symptoms but often face challenges due to the complexity of neuropathic pain. Neuromodulation is emerging as an important therapeutic approach for the treatment of neuropathic pain in patients unresponsive to common therapies, by promoting the normalization of neuronal and/or glial activity and by targeting cerebral cortical regions, spinal cord, dorsal root ganglia, and nerve endings. Having a better understanding of the efficacy, adverse events and applicability of neuromodulation through pre-clinical studies is of great importance. Unveiling the mechanisms and characteristics of neuromodulation to manage neuropathic pain is essential to understand how to use it. In the present article, we review the current understanding supporting dorsal root ganglia and spinal cord neuromodulation as a therapeutic approach for neuropathic pain.

## 1. Introduction

It is known that the sensation of pain has an important role in the maintenance of life, acting as a warning to remind us that something harmful is happening and that attention and action are needed to prevent further tissue damage [1,2]. However, when it exceeds the protective threshold and begins to cause harm, pain becomes distressing.

Nociceptors, specialized primary afferent neurons located in the peripheral nervous system (PNS), are adapted to sense danger, recognizing intense mechanical, thermal, and chemical stimuli which modulate ion channels, including sodium channels (Nav1.7, Nav1.8, and Nav1.9) [3] and/or transient receptor potential (TRP) channels [4,5], creating action potentials towards the spinal cord or trigeminal nuclei and relayed to the brain, to be translated and understood as pain. There are different modalities of pain, such as exacerbated pain (hyperalgesia), or responses to non-noxious stimuli, such as light touch, that can cause pain (allodynia) [6,7,8,9,10,11].

Injuries or diseases that affect the somatosensory system initially provoke a painful protective response in that specific innervated part of the body [12]. However, with persistence and recurrence, the nervous system responds inappropriately and excessively to damage, resulting in an imbalance of the somatosensory system and the generation of a state of chronic pain [12].

The classification of pain based on its mechanisms is a field in constant evolution. The current classification of pain by IASP (2017) is based on the pathophysiological mechanisms and separates pain into nociceptive, neuropathic, and the newest addition, nociplastic pain (which is still a concept to be settled) [13,14]. 

In nociceptive pain, the somatosensory nervous system functions normally, thus contrasting with neuropathic pain. It is the result of altered nociception or the activation of peripheral receptive terminals of primary afferent neurons in response to actual or potential damage to non-neuronal tissues [15,16,17,18]. It can be described as aching, throbbing, or pressure-like pain, and normally the patient only feels hypersensitivity around the area of the anatomical structure or where the acute injury is [14]. In addition to pain, the most concerning part of this condition is that it ends up affecting psychological and physical aspects of individuals, leading to depression, anxiety, insomnia, the emergence of cardiovascular diseases, hypertension, and even obesity [14,17]. This pain can be categorized as somatic or visceral nociception [14,19,20]. Somatic nociceptive pain involves the dermis, which, when stimulated, can cause burning, lacerating, and stinging pain (e.g., bone fracture, muscle spasm, burns, osteoarthritis) [14,20]. In contrast, visceral nociceptive pain is described as a diffuse and poorly localized sensation that can be diffuse in different areas due to the low density of visceral sensory innervation, but is normally present in the midline of the body, in the lower sternum or upper abdomen (e.g., renal or kidney stones, cirrhosis, pancreatitis, peptic ulcer) [14,20]. 

Nociplastic pain is described as an alteration in nociception (e.g., increased expression of sodium channels, cortical reorganization, immune system activation), increasing responsiveness within the central nervous system, without clear evidence of how or when the damage or disease (e.g., fibromyalgia, nonspecific back pain, bladder pain syndrome, irritable bowel syndrome, and temporomandibular disorder) led to this activation, and symptoms that end up affecting the psychological and physical conditions of patients [13,14,21,22]. This pain is described as a sharp, diffuse, or aching pain, comparable to neuropathic pain conditions; however, the pain pattern differs in having more sensitivity and hyperalgesia to mechanical stimuli than allodynia [14]. Currently, nociplastic pain is described as a broad and generic definition which may, in the future, change as studies advance, as neuronal plasticity is a condition that is not specific only to nociplastic pain, but can also be found in other types of pain, such as nociceptive or neuropathic pain [15,22]. 

Neuropathic pain is defined by the IASP as “pain caused by an injury or disease of the somatosensory nervous system” [14,23]. Regarding painful symptoms, individuals with neuropathic pain can present up to five different dimensions, namely (1) superficial burning pain, (2) deep pain, (3) paroxysmal pain, (4) evoked pain, and (5) dysesthesia or paresthesia [24,25]. In cases of central neuropathic pain, patients present a pattern of secondary hyperalgesia; that is, there is an increased sensitivity to pain outside the site of the injury [26]. These signs and symptoms, especially pain, significantly affect patients’ quality of life. Individuals with neuropathic pain may present a combination of positive phenomena (e.g., painful symptoms) and negative phenomena (e.g., neurological sensory deficits, motor deficits, and cognitive deficits) [27]. However, some patients may receive the term “mixed pain” [28], which occurs when different types of pain share a common characterization of clinical manifestation, overlapping each other. For example, individuals with a neuropathic disorder such as lumbar radiculopathy or cervical pain may experience nociplastic and/or nociceptive pain together [28,29,30]. However, this condition cannot happen the other way around. To meet the criteria and be considered neuropathic pain, the pain must be limited to a neuroanatomical distribution and not merely indicate a condition affecting the peripheral or central nervous system. This definition is necessary to avoid overriding other diagnoses. Additionally, if the pain spreads beyond the specific nerve area, it may develop and manifest as nociplastic pain along with neuropathic pain [18]. Some etiologies that may contribute to the development of neuropathic pain are summarized in Table 1 [12,24,26,31]. The IASP also classified pain syndromes that fall under chronic neuropathic pain (Table 2) [32,33].

Epidemiological data demonstrate that the prevalence of neuropathic pain in the general population is approximately 6.9–10% [24,34,35], with an estimated increase over the years due to risk factors (e.g., population aging and increased survival of cancer patients) [36]. In the United States, approximately 20 million people suffer from this condition [37]. Additionally, 20–25% of individuals with chronic pain have neuropathic pain [27].

As noted, it is of great importance to better understand how neuropathic pain occurs and the possible treatments that act and have a great effect on the neuronal component, as it is one of the main cellular types affected during this chronic pain. Neuromodulation methods target neuronal function; therefore, they represent possibilities for therapeutic approaches to control pain. The present narrative review simultaneously addresses DRG and spinal cord neuromodulation mechanisms applicable to reduce neuropathic pain in preclinical and clinical settings. We focus here on these two anatomical structures, since they interact strongly, bringing the context of the first step of peripheral primary neurons in communicating with the CNS within the spinal cord, which, to our knowledge, have not been addressed together in the literature on analgesic neuromodulation mechanisms.

## 2. Mechanisms of Neuropathic Pain

**Figure 1:**
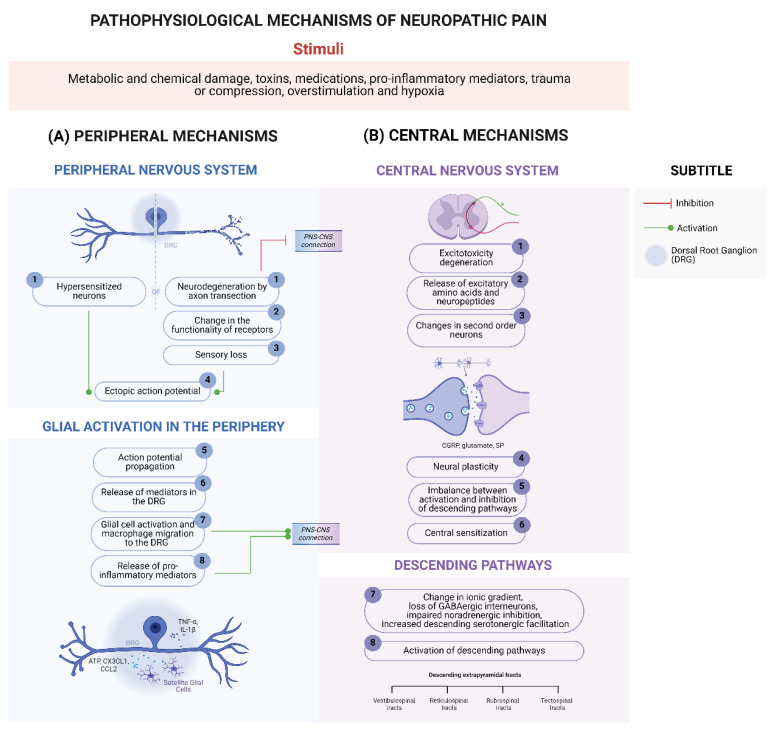
Pathophysiological Mechanisms of Neuropathic Pain. The initial damage to peripheral nerves occurs through varied noxious stimuli [39,40,41]. (**A**) Peripheral Mechanisms. (A1) In the PNS, this damage culminates in neurodegeneration due to the transection of neuronal axons [42], affecting the connection between the PNS and the CNS and (A2) the function of nociceptors, (A3) contributing to sensory loss [43]. (A1) Furthermore, primary sensory neurons can become sensitized, either to noxious stimuli or to the local inflammatory response [43,44]. (A4) In both cases, the persistence of the stimulus and the extent of PNS damage culminate in the generation of maladaptive repair, producing neuromas responsible for the ectopic action potential (summarized in Figure 2) [45,46]. (A5) Action potential propagation from peripheral nerves causes the (A6) release of several mediators in the DRG (e.g., ATP, CX3CL1, and CCL2), (A7) which activate satellite glial cells through specific receptors [47,48,49]. (A7) Taken together, the neuroinflammatory response initiated upon initial injury induces macrophage migration to the DRG [50]. (A8) The result is the release of pro-inflammatory mediators, such as TNF-α and IL-1β, which further amplify the sensitization and activation of nociceptor neurons causing pain [43]. (**B**) Central Mechanisms of neuropathic pain. (B1) With the persistence of the initial stimulus and the continuous input of afferent stimuli, the superficial neurons of the dorsal horn of the spinal cord also undergo degeneration due to glutamate-mediated excitotoxicity [51]. (B2) Furthermore, afferent fibers release excitatory amino acids and neuropeptides (e.g., calcitonin gene-related peptide, glutamate, and SP) into the dorsal horn of the spinal cord, (B3) promoting postsynaptic changes in second-order nociceptive neurons [50]. (B4) These changes result in neuronal plasticity, (B5) imbalance between the activation and inhibition of descending pathways, and (B6) central sensitization [52]. (B7,B8) As a result, there is abnormal entry of information into the cortex. Created using http://BioRender.com (accessed on 20 May 2024).

As seen previously, neuropathic pain can be divided according to the site of the injury or underlying disease. Injuries or diseases that affect the PNS alter signal transmissions from the periphery to the spinal cord through maladaptive plastic changes involving axonal sprouting and the formation of basket structures, causing neuropathic pain [26,38]. In addition, peripheral neuropathic pain also involves central sensitization mechanisms, since the damage caused to peripheral nerves generates persistent and dysregulated responses in the somatosensory system [26]. On the other hand, lesions or diseases that affect the CNS lead to amplified central processing due to a disinhibition at the spinal, thalamic, and cortical levels [26]. Next, we describe the peripheral and central pathophysiology of neuropathic pain. Figure 1 summarizes the main mechanisms involved in neuropathic pain.

### 2.1. Peripheral Mechanisms of Neuropathic Pain

The onset of peripheral neuropathic pain is due to the interaction between the peripheral terminals of C fibers and Aδ fibers (both related to pain processing) with different noxious stimuli, culminating in neuronal hypersensitivity [12]. Some examples of stimuli, as well as the mechanisms described below, are mentioned in Figure 1.

The initial noxious stimulus is capable of causing neurodegeneration, which culminates in an interruption between the PNS and the CNS and alteration of the ion channels and receptors expressed by nociceptive sensory neurons [65]. The same phenomenon can be observed in superficial neurons of the dorsal horn of the spinal cord [66]. These mechanisms contribute to the development of sensory loss, resulting in an area with reduced sensitivity to painful stimuli, whether thermal or mechanical [26].

**Figure 2:**
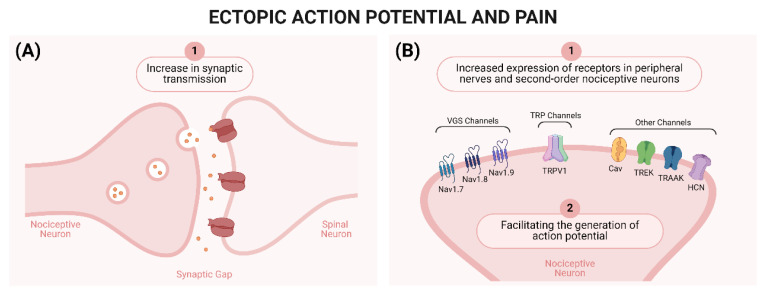
Ectopic Action Potentials and Neuropathic Pain. (**A**) (A1) Ectopic action potentials are generated through an increase in synaptic transmission between nociceptive neurons and spinal neurons [43]. (**B**) (B1) Taken together, the increased expression of voltage-gated sodium channels (VGSC; Nav.1.7, Nav 1.8, and NaV1.9) [53,54,55,56], calcium channels (Cav), potassium channels (TREK and TRAAK), cyclic nucleotide gated channels (HCN) [57,58,59,60], and transient receptor potential (TRP; TRPV1) [61,62] also contribute to the generation of ectopic action potentials. (B2) Plastic changes in nociceptor neurons, including the enhanced expression and activity of these channels, facilitate the generation of action potentials, resulting in neuronal sensitization [63,64]. Created using http://BioRender.com (accessed on 20 May 2024).

Consequently, areas affected by sensory loss and sensitized sensory neurons begin to generate ectopic action potentials [67,68]. These action potentials can occur at the injured site or at more distant locations, such as the DRG [68,69], as they are caused by two different factors: (1) increased synaptic transmission [26] and (2) increased expression of channels important for nociceptive signal transmission [39,43,70,71]. Similar changes can occur in second-order nociceptive neurons after central lesions, culminating in the development of central neuropathic pain. Figure 2 summarizes the role of ectopic action potentials in neuropathic pain.

Another class of channels of great importance for the development of neuropathic pain are the TRP channels, especially the vanilloid subtype 1 (TRPV1) channels [26]. These channels will be activated by various stimuli (physical and chemical), which contribute to the depolarization of the membrane of sensory neurons, the activation of VGSC, and the generation of ectopic action potentials [4,43] (Figure 2).

The locally initiated inflammatory response also contributes to the development of neuropathic pain. Cells recruited to the injured site, such as neutrophils, macrophages, lymphocytes, and mast cells, release substances (e.g., prostaglandins and pro-inflammatory cytokines) that sensitize peripheral sensory neurons [26,72].

Previous studies have shown that glial activation is also involved in sensitization in cases of neuropathic pain. Satellite glial cells are found in the DRG near the central terminals of afferent nerves that have been peripherally injured [73]. DRG cells release mediators chemoattracting macrophages to this site where neurons, satellite glial cells, and macrophages interact toward nociceptive signaling to the spinal cord [74].

### 2.2. Central Mechanisms of Neuropathic Pain

According to the IASP, central sensitization can be defined as “an increase in the responsiveness of nociceptive neurons in the CNS to their normal or subliminal afferent input” [75]. The central mechanisms of neuropathic pain are described in Figure 1.

Central sensitization targets central and supraspinal nociceptive pathways through persistent and intense nociceptive stimuli [12]. These stimuli promote the release of excitatory amino acids and neuropeptides in the dorsal horn of the spinal cord, promoting postsynaptic changes in second-order nociceptive neurons [74], such as ion channel phosphorylation (e.g., ionotropic and metabotropic glutamate channel receptors phosphorylation or VGSC) [76], changes in calcium permeability, and modifications in gene expression [77]. All these changes result in neuronal plasticity that culminates in an imbalance between the activation and inhibition of descending pathways, thus generating a state of central sensitization [77].

**Figure 3:**
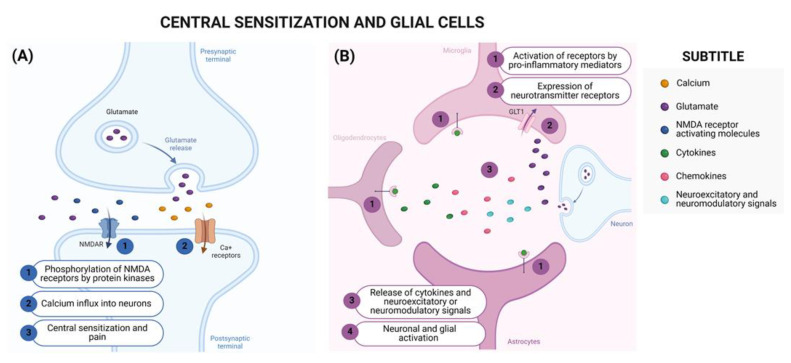
Central Sensitization and Glial Cells. (**A**) (A1) N-methyl-D-aspartate (NMDA) receptors contribute to central sensitization and their trafficking to the membrane is regulated by protein kinase C-dependent phosphorylation. (A2) The activation of NMDA receptors promotes the influx of calcium into neurons and the activation of intracellular signaling pathways, (A3) contributing to nociceptive neurotransmission [87,88]. (**B**) (B1) Glial cells, such as astrocytes, microglia, and oligodendrocytes [89], can be activated through Toll-like receptors (TLRs) and cytokines (IL-1β, TNF-α, and IL-6) produced during the development of the nociceptive response [90]. (B2) Furthermore, glial cells express several receptors for neurotransmitters, which can be activated through synaptic transmission [91]. (B3) When activated, these cells can further release cytokines (IL-1β, TNF-α, and IL-6) [92,93] and pro-inflammatory chemokines (CCL2 and CX3CL1) [94,95], or neuroexcitatory or neuromodulators signals (ATP, glutamate, and SP) [96], (B4) culminating in enhanced neuronal and glial activation [91]. Created using http://BioRender.com (accessed on 20 May 2024).

The participation of N-methyl-D-aspartate (NMDA) receptors in central sensitization after nerve injury can be highlighted [26,78] due to their participation in nociceptive transmission and synaptic plasticity [79]. Furthermore, the activation of glial cells (astrocytes, microglia, and oligodendrocytes) in the CNS following peripheral or central injuries also contributes to the development of central neuropathic pain [74]. Microglial activation occurs not only in the spinal cord, but also at supraspinal levels [74,80,81] such as in the thalamus, sensory cortex, and amygdala, promoting increased synaptic plasticity in the somatosensory cortex and, consequently, hypersensitivity [80,81]. Figure 3 summarizes the involvement of NMDA receptors and glia cells in neuropathic pain.

As a result of these events, there is a reorganization of the descending pathways responsible for modulating nociceptive input in the spinal cord [26,79]. Under physiological conditions in the spinal cord, there is a balance between the activation and inhibition of pro- and anti-nociceptive pathways, with transmission inhibition prevailing [26,79]. However, in pathological conditions, such as in cases of neuropathic pain, activation becomes predominant over inhibition [26,79]. This phenotypic switch occurs through alterations in ion gradients [82], the loss of GABAergic interneurons [83], impaired noradrenergic inhibition [84], and increased downward serotonergic facilitation [85,86], all of which, together with the altered peripheral input, contribute to abnormal information received by the brain.

## 3. Current Guidelines for the Treatment of Neuropathic Pain

**Figure 4:**
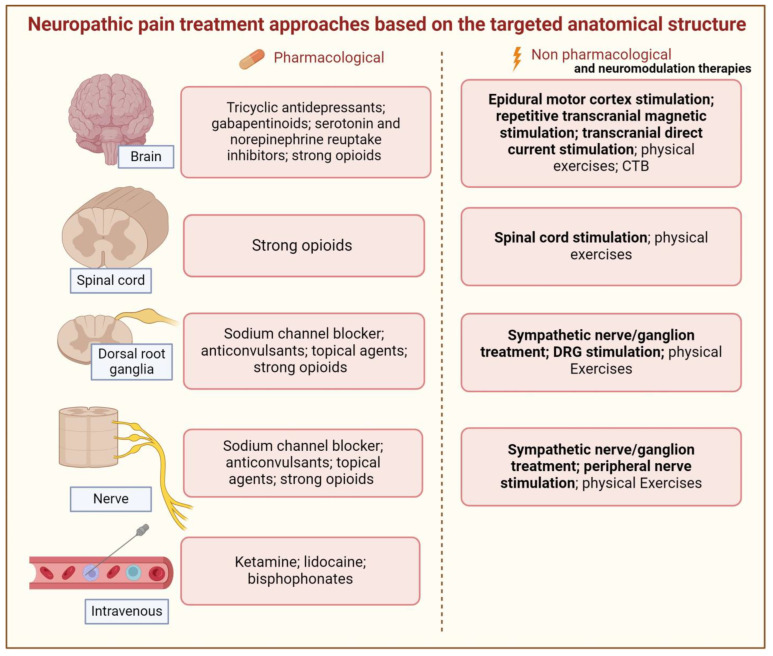
Neuropathic pain treatment approaches based on the targeted anatomical structure. Neuromodulation therapy approaches and site of approach are highlighted in bold. CBT (cognitive behavioral therapy). Created using http://BioRender.com (accessed on 30 April 2024).

Therapeutic approaches for the control of neuropathic pain have limitations and vary in the quality of evidence, ranging from low to high level, and are directed toward the improvement of symptoms and, in some cases, the improvement of etiological causes. Currently, treatment is divided into pharmacological and non-pharmacological approaches [33,37]. Figure 4 and Table 3 summarize the main current treatments (pharmacological and non-pharmacological) for improving neuropathic pain. An interesting point is the application of physical exercise to control neuropathic pain. In general, studies will differentiate between aerobic and anaerobic exercises, and how each type of muscle contraction acts on pain. We suggest some review articles on this topic [40,41,97,98,99,100].

Current pharmacological approaches consist of the medical prescription of seven main classes of drugs: (1) serotonin- and noradrenaline modulating antidepressants, (2) sodium-blocking anticonvulsants, (3) calcium-modulating anticonvulsants, (4) tramadol, (5) opioids, (6) capsaicin, and (7) local anesthetics [79]. These classes are subdivided into first-, second- and third-line drugs according to the Neuropathic Pain Special Interest Group (NeuPSIG). Non-pharmacological treatments include interventional therapies (e.g., peripheral nerve block, sympathetic nerve or ganglion treatment, and peripheral and central neurostimulation)—indicated in cases of untreated neuropathic pain—physical therapies (e.g., physiotherapy), and psychological therapies [33].

## 4. Neuromodulation Therapies

Neuromodulation denotes a scientific field of medicine and bioengineering that encompasses implantable and non-implantable technologies and electrical or chemical interventions aiming to improve the quality of life and the functioning of the human nervous system, including during neuropathies. Emerging as a therapeutic alternative, neuromodulation acts by stimulating, modifying, regulating, or inhibiting the activity of neurons in the nervous system. The process is reversible and can affect both the PNS and the CNS. Electrical neuromodulation is routinely associated with brain stimulation, spinal cord stimulation, and peripheral nerve stimulation. Meanwhile, chemical neuromodulation is linked to the implementation of drug delivery systems or other substances via epidural or intrathecal routes. Neuromodulation in general occurs through the implantation of neural stimulators or micro pumps for infusion. Its use is regulated in humans and is associated with chronic diseases, being applicable in the treatment of psychiatric disorders, epilepsy, spatial dysfunctions, and chronic pain management [55].

The use of neuromodulation to treat pain began in 1967, when Wall and Sweet published a case report involving seven patients suffering from chronic peripheral pain caused by various conditions. The authors reported that the electrical stimulation of peripheral nerves significantly reduced pain in these patients; however, they noted that the analgesic effect of the technique diminished over time, with its duration reported to be within months (specific time frame not provided) [56]. Subsequently, based on Wall and Sweet’s report, Shealy and colleagues conducted a new case study involving 10 patients who reported experiencing pain secondary to various diseases. The authors performed electrical stimulation of the peripheral nerves and reported an analgesic effect in all patients [63]. It is believed that this effect may be due to the premise that the stimulation of Aβ fibers from fast-conducting mechanoreceptors may prevent slow-velocity signal conduction nociceptive signals transmitted by Aδ and C fibers from reaching higher brain centers, thus resulting in analgesia [64].

According to the International Neuromodulation Society, neuromodulation therapy is currently indicated as a fourth-line treatment of neuropathic pain when patients fail to achieve satisfactory levels of relief using other conventional pharmacotherapies, but prior to low-dose opioids. This recommendation is based on the premise that neuromodulation therapies are employed for chronic pain conditions that are persistent and resistant to standard pharmacological therapies (International Neuromodulation Society—accessed on 28 May 2024). The techniques share similar mechanisms of action that interfere with the function of the nervous system, affecting multiple structures and resulting in analgesia. However, each modality seems to have its singular mechanism of action.

As previously mentioned, pain occurs through the activation of cortical areas of the CNS, which are typically triggered by the detection of a noxious stimulus. The mechanism of neuromodulation therapies differs according to the region stimulated, and their analgesic effect depends on the duration and intensity of the stimulus. The main application sites for neuropathic pain therapy are the peripheral nerves, DRG, and spinal cord. Neuromodulation can target various areas, including the cerebral cortex, spinal cord, dorsal root ganglia, peripheral nerves and their receptive fields, depending on the patient’s symptomatic profile and anatomical considerations. For instance, patients experiencing persistent headaches may be recommended for intracranial stimulation, while those with focal neuropathic pain are advised to undergo peripheral or spinal nerve stimulation [64].

Regarding the spinal cord, the primary method of stimulation is through intradural spinal cord stimulation (SCS), which involves the implantation of a subcutaneous pulse generator (associated with the dermatomal areas where pain is anatomically represented) connected to electrodes placed in the epidural space towards the dorsal region of the spinal cord, which inhibits pain signaling in the spinothalamic tract. Its mechanism of action is based on the activation of Aβ fibers and inhibitory interneurons, disrupting the afferent sensory input from the DRG and releasing inhibitory neurotransmitters (e.g., GABA) in the spinal cord and CNS [103]. Essentially, SCS devices distribute electrical impulses through electrodes, and stimulate Aβ fibers of the dorsal column with tonic electrical impulses, inducing paresthesia in the body region represented by the stimulated sensory fibers. Aβ fibers then synapse at the spinal level with smaller nociceptive neurons, and the activation of these synapses inhibits the transmission of the nociceptive signal, effectively reducing the pain sensation experienced by the patient, thus explaining one of the mechanisms of action of SCS [104]. Its application is mainly directed towards neuropathic pain, as it exhibits a better effect compared to analgesia obtained in types of pain [84,85]. The intensity and duration of the impulse are crucial in the treatment of the condition. Neuromodulation therapies involving high-frequency (HF) SCS utilize short duration (30 μs), high frequency (10,000 Hz), and low amplitude (1–5 mA) pulses without paresthesia, primarily applied at T8–T11 spinal levels and in cases of lumbar or lower extremity pain. Conversely, burst spinal cord stimulation employs a low-energy mode that uses a pulse consisting of five consecutive waves with a fixed internal frequency (500 Hz) and pulse width (1 ms interval) 40 times per second. In both the high-frequency and burst methods, pre-programmed circuits (i.e., programmed for only one configuration) are inserted. In contrast to these, closed-loop spinal cord stimulation involves electrodes capable of monitoring neuronal electrical activity in the region, enabling the device to titrate the intensity, frequency, and duration of the pulse according to the microenvironment. DRG stimulation is theoretically more selective compared to spinal cord stimulation. It is primarily applied to patients with pain in the distribution of discrete dermatomes from T10 to S2. It has the ability to act directly on nociceptive afferent neurons, “normalizing” their electrical activity without influencing other systems. The stimulation of nerve endings uses the same principles as spinal stimulation and DRG, but is believed to be less effective in treating PN. We will next discuss preclinical and human studies bringing about major advancements in neuromodulation therapies for neuropathic pain treatment, and focusing on the DRG and spinal cord levels, since we observed a gap in the literature reviews addressing neuromodulation therapies at these structural levels.

## 5. Neuromodulation Therapies Targeting DRG and the Spinal Cord in the Management of Neuropathic Pain

### 5.1. Neuromodulation Therapies at the DRG Level

Preclinical studies employing neuropathic pain models are critical for basic research to promote a better understanding of how the mechanisms of neuromodulation therapies target pain and other symptoms of the disease. Rodent models in preclinical studies apply various forms of nerve injury to induce neuropathic pain, including peripheral nerve injury [chronic constriction injury (CCI), partial nerve ligation (PNL), nerve root ligation (NRL), spared nerve injury (SNI), etc.], toxic agents (induced by chemotherapy, streptozotocin, and alcohol), and infectious agents (e.g., post herpetic neuralgia) as well as targeting specific nerve branches to mimic diseases such as trigeminal neuralgia and orofacial pain [57,58]. Each of these models has a particular methodology, behavioral manifestations, limitations, and advantages. However, the creation of these models has substantial contributions to the understanding of chronic pain and underlying peripheral and central pathophysiological mechanisms in neuropathic pain.

DRG neurons are pseudo-unipolar cells that bridge the gap between peripheral tissues and the spinal cord, delivering their products through their two nerve endings, with implications in both the PNS and CNS [59,60]. Using a model of tibial nerve injury in rats and DRG stimulation at the L_4_ level, replicating a clinically used load (20 Hz, 150-ms pulse width, current at 80% of motor threshold), it was shown that cold and mechanical hyperalgesia and allodynia were reduced [61]. In subsequent studies that further explored its mechanisms, it was demonstrated that DRG neuromodulation therapy [high-frequency electrical nerve stimulation (HFES)] applied in in vitro (10 kHz, 2 mA) or in vivo (20.6 kHz) systems was able to reduce the production of high-mobility group Box-1 (HMGB1), CGRP, and SP peripherally as well as reduce pain, thus indicating reduced nociceptor neuron activity, suggesting that HFES may reset sensory neurons to a less pro-inflammatory state [62], highlighting a potential mechanism for the treatment of neuropathic pain. Moreover, in rats with SNI procedure, high-voltage pulsed radiofrequency (HVPRF) in the DRG (L_5_; 45–100 V) reduced pain and induced autophagy in spinal cord microglia, as well as reduced TNF-α and increased IL-10 were observed in the dorsal horn of the spinal cord, with additional improvements in DRG morphology [44]. Similar to these findings, DRG electrical stimulation (GFS—ganglionic field stimulation) could attenuate the blood oxygen-level dependent (BOLD) signal response to acute noxious stimulation in brain regions associated with painful stimuli in a rat model [105].

Regarding additional data applying direct peripheral nerve stimulation, when the sciatic nerve of rats with neuropathic pain (L_5_ NRL) was stimulated with 2–20 Hz frequencies, neuromodulation therapy could reduce pain-like behavior. This was explained by the reduction in spinal cord inflammatory proteins (NF-κB, TNF-α, IL-1β, and IL-6) as well as the inhibition of astrocytes and microglia. Furthermore, neuromodulation therapy increased the activation of the analgesic descending serotoninergic pathway [106]. Finally, in mice with SNI, TENS stimulation (100 HZ) led to decreased hyperalgesia by inhibiting glial activation, MAP kinase activation, PKC-γ, and p-cyclic-AMP response element binding (CREB) expression, and proinflammatory cytokines, with an additional increase in spinal cord opioid receptors [47], thus providing insights into how neuromodulation therapies applied on DRGs and related spinal nerves can reduce neuropathic pain.

DRG neuromodulation therapy emerged later than traditional SCS as an alternative approach. DRG stimulation is effective across a range of frequencies, with 20 Hz being the default frequency [59]. In one of the first clinical trials evaluating DRG stimulation for the management of neuropathic pain, the patient population presented with neuropathies associated with intractable pain in the back and lower limbs. They received implants placed epidurally near the DRGs, and neuromodulation reduced pain by 56% at 12 months post-implantation, as well as pain in the spine and proximal and distal lower limbs, coupled with reports of improvements in quality of life and mood [48]. Also, dorsal root ganglion stimulation (DRGS) presented the ability to restore the alterations (correlated with pain intensity) of patients’ laser-evoked potentials (LEPs), a recognized method for detecting subcortical lesions of the A-delta fiber pathways, known to be pain- and temperature-transmitting pathways [60]. Furthermore, in a prospective randomized trial (12 month follow-up), patients diagnosed with CRPS (complex regional pain syndrome) or causalgia in the lower limbs and receiving DRG stimulation (19–20 Hz) reported a 50% or greater decrease in visual analog scale (VAS) score from pre-implant baseline. Additionally, patients did not report any stimulation-related neurological deficits. Importantly, paresthesia-free patients had equivalent or improved outcomes for pain parameters, quality of life, and mood state when compared to subjects with paresthesia-present stimulation [49,50]. DRG stimulation-related paresthesia was less frequent and intense on average, with a smaller footprint on the body and less dependence on positional changes compared to tonic SCS. These results suggest that the presence of stimulation-induced paresthesia during DRG stimulation is not as critical to the success of therapy compared to tonic SCS [104]. Patients with chronic discogenic low back pain due to failed back surgery syndrome have benefited from DRG stimulation at L_2_ or L_3_, reporting 50% or better relief of low back pain [107].

Piedade et al. tested low-frequency DRG stimulation in patients with scores above 12 in a PainDetect questionnaire (CRPS, postsurgical pain, and intercostal neuralgia), and showed no statistical difference in pain intensity relief when compared to the classic 20 Hz frequency; however, the authors support the idea that clinicians should offer patients different stimulation frequencies under DRG stimulation, aiming for higher efficacy [108]. Additionally, the use of burst SCS microdosing (alternating stimulation-on and stimulation-off periods) has been shown to be equivalent to that used for standard burst stimulation in neuropathic pain patients with back and leg pain [52].

### 5.2. Neuromodulation Therapies Applying SCS

Previous preclinical studies have demonstrated that tonic SCS can stimulate spinal interneurons, which has been attributed to the closure of the spinal cord gate (gate control theory), which has been postulated to be due to the release of GABA, thus inducing analgesia [109,110,111]. However, recent preclinical studies also point to novel potential effects of SCS on the spinal cord. It is known that the activation of glial cells in the spinal cord plays a key role in the chronicity of neuropathic pain, since these cells secrete a huge range of nociceptive molecules accounting to a high activation of dorsal horn neurons. In turn, dorsal horn neurons also release neurotransmitters and neuropeptides that participate in the maintenance and potentiation of nociceptive signaling, as well as neuronal plasticity [112]. It is believed that neuromodulation therapies in the spinal cord may decrease glial cell activity, as classical markers of these non-neuronal cells decreased upon neuromodulation interventions [47,90], and consequently, NF-κB activation and levels of downstream inflammatory molecules, including TNF-α, IL-1β, and IL-6, are also reduced [106]. Neuromodulation therapies can also directly reduce neuronal activity in both the brain [91] and the spinal cord. Therefore, by restraining glial cell activation and reducing neuronal activity, neuromodulation can reduce pain-like behaviors.

In the initial data from studies investigating neuromodulation therapies using SCS implantable devices, PNL mice stimulated for 30 min (50 Hz; pulse width 0.2 ms) showed a notable reduction in pain [44,92,109]. In a rat model of CCI, SCS significantly reduced pain and microglial activation in the spinal dorsal horn, which were associated with reductions in sensory neuron-derived colony-stimulating factor 1 (CSF1) levels in the spinal dorsal horn and dorsal roots but not DRG [93]. Thus, evidence of the effects of SCS may be restricted to CNS, suggesting its use for neuropathies of central origin. Subsequent studies demonstrated that increases in SCS duration and amplitude (4–60 Hz frequency) cause further reduction in pain and glial cell activation in SNI mice. A time-dependent effect of SCS as well as opioid receptor-dependent mechanisms was observed, since 4 Hz SCS resulted in the activation of μ-opioid receptors, whereas 60 Hz SCS was involved the activation of δ-opioid receptors [90,94]. Additionally, paresthesia-free analgesia may not be limited to ultra-high frequency SCS (10 kHz) but may be achieved by lower frequencies of SCS (200–1200 Hz; 1–0.2 msec; 120 min) that achieved similar effects if the electrical dose was optimized in SNL rats [95]. Since traditional SCS is related to uncomfortable paresthesia and may present suboptimal efficacy, accurate dose adjustment may be a crucial aspect for the success of neuromodulation therapy.

Another interesting point is that the production of specialized pro-resolving mediators (SPM) may be impaired in neuropathic pain. These mediators are responsible for maintaining the balance of the immune system and regulating nociception [113,114]. In this sense, Tao et al. demonstrated in a recent study that spinal electrical stimulation (1 kHz) can restore the production of resolvin D1 in the cerebrospinal fluid and consequently reduce the increased production of IL-1β and mechanical and cold allodynia in SNI rats [115]. Resolvin D1 is an analgesic pro-resolving lipid mediator that downregulates nociceptor neuron activity and neuroimmune interactions [96].

Data in the literature from studies using SCS showed the use of different protocols (individual or combined) and techniques in neuropathic pain conditions. Tonic SCS can range from 30 to 80 Hz, 100 to 500 µs of pulse width, and an amplitude above sensory threshold [116,117]. High-frequency CS and burst SCS commonly use amplitudes below the sensory threshold, at which the patient does not experience paresthesia during stimulation.

For conditions such as chronic intractable pain, clinical trial results point to similar clinical outcomes achieved in burst SCS when performing lead placement with imaging references either using paresthesia mapping or anatomical landmark-based approaches [118,119]. In a randomized, placebo-controlled study, anodal trans-spinal Direct Current Stimulation (tsDCS; 2.5 mA for 20 min) reduced the perception of pain in the foot, most pronounced during the 30 min after its cessation, and in the hand. However, analgesia was less pronounced in the hand, demonstrating its potential applicability for neuropathic pain management, although with tissue specificities [120]. Conventional SCS (low-frequency SCS) has been variably defined as frequencies from 30 to 200 Hz. The percentage of pain relief in subjects with CRPS-I is reduced after SCS but not DRG stimulation at post-permanent implant follow-up visits (especially at 9–12 months) [121]. However, when DRG stimulation and SCS implants (5-pulse “clustered” protocol waveform at 80 Hz) are associated in patients with CRPS, sustained analgesia is achieved for up to 3 years, demonstrating the potential efficacy of the combined intervention [122]. Furthermore, after association with peripheral nerve field stimulation (PNFS), the effects of SCS are improved, promoting significant pain reduction and improving quality of life [123]. Interestingly, sub-perception SCS neuromodulation (≤1.2 kHz) enables the rapid onset of analgesia in patients with chronic pain. In fact, sub-perception SCS is noninferior to sub-perception approaches, is safe and effective in subjects with extreme physical disability, and has previously been implemented for neuropathic pain [124].

SCS (40 Hz) in patients with CRPS led to a significant reduction in pain, hyperalgesia, and allodynia [125]. A recent well-designed study demonstrated, through the evaluation of dynamic brain imaging responses [positron emission tomography (PET)-computed tomography (CT)], that SCS with higher frequencies (4000 vs. 40 Hz) provided additional effects in the nociceptive and affective areas of pain processing, especially thalamic areas in patients with intractable lumbar neuropathic pain, highlighting the potential mechanisms of SCS intervention [126]. In patients with painful diabetic neuropathy (PDN) exposed to high-frequency SCS implants (30-ms pulses at 10,000 Hz), improvements in lower-extremity pain, weakness, and positive sensory symptoms were observed in a 12-month follow up [127]. This study corroborates a prior one that showed that SCS (frequency not reported) significantly reduced pain and improved quality of life in patients with refractory PDN [102], thus supporting the role of SCS in the treatment of PDN. In a sample of patients with neuropathic chronic low back pain (LBP), a paresthesia-free SCS waveform technique promoted the significant and successful attenuation of the VAS and brief pain inventory (BPI) score after 26 weeks of intervention. Improvements in patients’ reported quality of life were also notable [128]. Benefits have also been demonstrated for elderly people with postherpetic neuralgia receiving both pulsed radiofrequency (50 Hz, 0.5 V) and short-term SCS (stSCS; 20–80 Hz; 1–3 V; and pulse width, 210–450 μs); however, the latter provided a better and longer-lasting analgesic effect compared to pulsed radiofrequency [129].

## 6. Discussion and Conclusions

**Figure 5:**
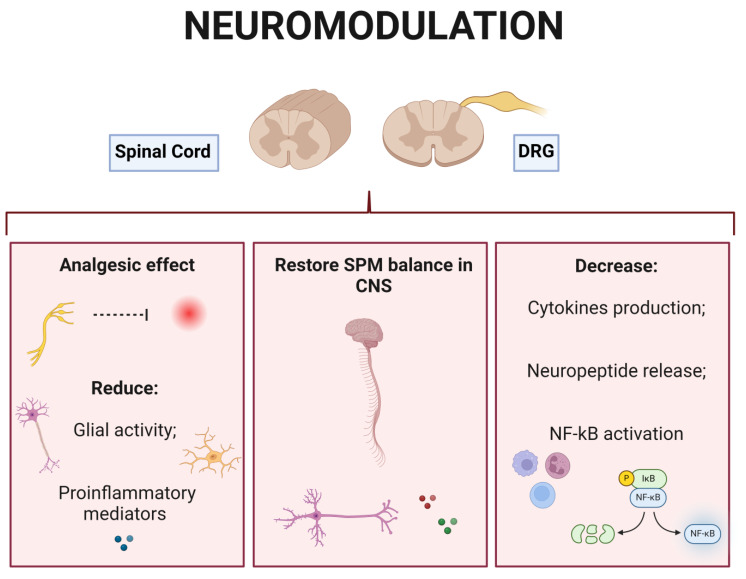
Neuropathic pain mechanisms that are targeted by neuromodulation therapies. Created using http://BioRender.com (accessed on 29 May 2024).

An understanding of neuropathic pain involves not only the detection of a simple sign of tissue damage, but also the complex interaction among biological, social, and psychological components. Neuropathic pain is characterized by an amplification and dysfunction of the signals involved in the nociceptive process in the PNS and CNS. Neuronal hyperexcitability, central sensitization, neuroinflammation, and maladaptation are the main mechanisms involved in the genesis of neuropathic pain. The variety of etiologies underlying this condition makes its management even more complex. Neuromodulation adds possible therapeutic approaches to effectively treat neuropathic pain. SCS can now be seen as a fundamental non-opioid choice for chronic neuropathic pain refractory to pharmacotherapy. New recent options for the application of neuromodulation therapies, including DRG stimulation, and high-frequency and burst SCS can now offer different options aiming at personalized and tailored treatment strategies for patients. Data from animal and human studies suggest potential benefits of neuromodulation targeting the DRG and spinal cord. The current understanding indicates that, in the DRG and spinal cord, the mechanisms of neuromodulation therapies for the treatment of neuropathic pain include several molecular and tissue effects. Briefly, the mechanisms of neuromodulation therapies within the DRG and spinal cord involve (a) decreased production of hyperalgesic cytokine (e.g., TNF-α, IL-1β, IL-6, and HMGB1), neuropeptide release (CGRP and SP), and NF-κB activation in the DRG; (b) induction of opioid signaling and activation of descending analgesic pathways within the spinal cord; (c) reduction of glial cell activity and pro-inflammatory mediators in the spinal cord; and (d) increase of SPM such as resolvin D1 produced in the CSF, reducing the production of neuroinflammatory cytokines such as IL-1β. These mechanisms are summarized in Figure 5.

Therefore, it is likely that a better understanding of tissue-dependent molecular and cellular mechanisms can help to precisely select the neuromodulation approach to reach better and personalized treatment. Basic, translational, and clinical studies are crucial for a better comprehension of the neuromodulation effectiveness, adverse events, and applicability. Preclinical studies remain crucial to elucidate the mechanisms of action of neuromodulation, and clinical trials, with large sample sizes and gold-standard characteristics, including randomization, blindness, and placebo control, are needed to determine the magnitude of effect of neuromodulation therapies on neuropathic pain management.

## Figures and Tables

**Table 1:** Etiologies of neuropathic pain.

Etiology	Pain Syndrome
Mechanics (trauma)	Post-surgical neuropathy
	Cervical and lumbar radiculopathy
	Cancer
	Traumatic nerve injury
	Spinal cord injury
	Amputation
Metabolic Disorders	Diabetic neuropathy (insulin-dependent and non-insulin-dependent)
	Vitamin B12 and folate deficiency
	Hypothyroidism
Inflammatory Disorders and Viral Infections	Postherpetic neuralgia
	HIV neuropathy
	Trigeminal neuralgia
	Chronic inflammatory demyelinating polyneuropathy
	Leprosy
Toxicity	Chemotherapy-induced peripheral neuropathyAlcoholic neuropathy
Radiation	Post-radiation neuropathy
Heredity	Charcot–Marie–Tooth disease
	Fabry disease
Autoimmune Diseases	Guillain–Barré Syndrome
	Multiple sclerosis
Unknown	Idiopathic neuropathy

HIV: human immunodeficiency virus.

**Table 2:** Chronic neuropathic pain syndromes according to their peripheral or central origin [32,33].

Origin	Pain Syndrome
Peripheral	Trigeminal neuralgia
	Chronic neuropathic pain after peripheral nerve injury
	Painful polyneuropathy
	Postherpetic neuralgia
	Painful radiculopathy
Central	Chronic central neuropathic pain associated with spinal cord injury
	Chronic central neuropathic pain associated with brain injury
	Chronic central pain after stroke
	Chronic central neuropathic pain associated with multiple sclerosis

**Table 3:** Therapeutic approaches for the control of neuropathic pain. Pharmacological and non-pharmacological options currently available are shown.

**Pharmacological Approaches Investigated in Studies**			
**Pharmacological Class (Drug)**	**Recommendation**	**Mechanism**	**References**
**First-line drugs**			
**TCA (amitriptyline)**	All types of NP	Modulate the activity of descending control systems (inhibit serotonin and noradrenaline reuptake from presynaptic terminals and inhibit cholinergic, adrenergic, and histaminergic receptors)	[42,51]
**Gabapentinoids (gabapentin, pregabalin)**	All types of NP	Attenuate central sensitization (brainstem and higher brain centers) by acting on Ca^2+^ channels (reduce Ca^2+^ influx)	[37,42,45]
**SNRI (duloxetine, venlafaxine)**	All types of NP data	Affect the reuptake of norepinephrine and serotonin by modulating the activity of the downward control systems	[37,42]

**Sodium channel blocker anticonvulsants (Carbamazepine, oxcarbazepine)**	Trigeminal neuralgia	Act on the peripheral mechanisms that generate pain	[37,42]
**Second-line drugs**			
**Weak opioid agonists (tramadol)**	All types of NP data	Activate inhibitory pre- and postsynaptic MORs receptors	[37,42]
**Topical agents (capsaicin, lidocaine)**	Peripheral NP and postherpetic neuralgia	TRPV1 channel agonist	[37,42]
**Third-line drugs**			
**Strong opioid agonists (morphine,** **oxycodone)**	All types of NP data	Activate inhibitory MORs located in the spinal cord, brain, and periphery (complexity of follow-up and adverse side effects of drugs of abuse)	[37,42]
**Botulinum toxin A** **(BoNTA)**	SCI and peripheral NP	Dose-dependent toxic action involving chemodenervation, inhibiting acetylcholine release and leading to functional denervation	[33,46]
**Infusion (IV) therapies** **(ketamine, lidocaine,** **bisphosphonates)**	CRPS, fibromyalgia, and traumatic spinal cord injury	Antagonism of NMDA receptor, exerting its effect through the modulation of the transmission of ascending nociceptive information and descending inhibitory pathways	[33,46]
**Non-pharmacological approaches investigated in studies**			
**Type**	**Recommendation**	**Mechanisms**	**References**
**Interventional therapies**			
**Sympathetic nerve/ganglion treatment**	Intractable NP	Blockage, neurolysis, or ablation	[33,42]
**Peripheral nerve stimulation**	Intractable lumbar NP	Peripheral and central actions, via modulation of inflammation, autonomic nervous system, and endogenous pain inhibition pathways	[33,101]
**TENS**	Intractable NP	Activation of large-diameter non-noxious afferents resulting in the release of inhibitory neurotransmitters which reduce activity in second-order nociceptive transmission cells	[74,102]
**DRG stimulation**	Refractory CRPS and lower limb causalgia	Stimulation of the post-synaptic activation of pain-gating circuitry in the DRG and modulation of the intrinsic excitability of DRG neurons.	[74,102]
**SCS**	Diabetic neuropathy, refractory ND, and fibromyalgia	Administration of electrical impulses capable of suppressing central neuronal hyperexcitability	[74,102]
**Epidural motor** **cortex stimulation**	Intractable NP	Endogenous opioid and cannabinoid systems via descending inhibitory pathways	[74,102]
**rTMS**	Intractable NP and PLP	Production of electrical currents in the cortex through a transient magnetic field	[74,102]
**tDCS**	Intractable NP, diabetic neuropathy, traumatic spinal cord injury, and fibromyalgia	Modulates axonal membrane potential	[74,102]
**Physical therapies**			
**Physical exercises**	Peripheral NP	Inducing opioid and cannabinoid pathways, increasing the levels of anti-inflammatory cytokines and neurotransmitters (e.g., serotonin and GABA), and decreasing growth factors such as NGF and BDNF, which results in the inhibition of glial cells in the spinal cord	[33,53]
**Psychological therapies**			
**CBT**	Chronic NP, diabetic neuropathy, cancer- associated ND, or HIV infection	Improvement in mood and diagnostic catastrophizing	[33,42]
**Associated interventions with exercise and psychological therapy** (**mediation, CBT**)	Diabetic neuropathy	Summed effects presented in the two descriptions above	[33,54]

TCA (tricyclic antidepressants); NP (neuropathic pain); SNRI (serotonin and norepinephrine reuptake inhibitors); IV (intravenous); CRPS (complex regional pain syndrome); NMDA (N-methyl-D-aspartate); SCI (spinal cord injury); TENS (transcutaneous electrical nerve stimulation); DRG (dorsal root ganglion); SCS (spinal cord stimulation); rTMS (repetitive transcranial magnetic stimulation); tDCS (transcranial direct current stimulation); CBT (cognitive behavioral therapy); MORs (opioid μ-receptors); TRPV1 (transient potential receptor vanilloid 1); Ca^2+^ (calcium); PLP (phantom limb pain); GABA (gamma-aminobutyric acid); HIV (human immunodeficiency virus).
